# Protective Effects of Bifidobacterium on Intestinal Barrier Function in LPS-Induced Enterocyte Barrier Injury of Caco-2 Monolayers and in a Rat NEC Model

**DOI:** 10.1371/journal.pone.0161635

**Published:** 2016-08-23

**Authors:** Xiang Ling, Peng Linglong, Du Weixia, Wei Hong

**Affiliations:** 1 Department of Neonatology, Children’s Hospital of Chongqing Medical University, Ministry of Education Key Laboratory of Child Development and Disorders, China International Science and Technology Cooperation Base of Child Development and Critical Disorders, Chongqing Key Laboratory of Pediatrics, Chongqing, China; 2 Department of Gastrointestinal Surgery, The First Affiliated Hospital of Chongqing Medical University, Chongqing, China; 3 Department of Kidney Immunology, Children’s Hospital of Chongqing Medical University, Chongqing, China; 4 Department of Pediatrics, Second People’s Hospital of Chengdu, Chengdu, China; SRI International, UNITED STATES

## Abstract

Zonulin protein is a newly discovered modulator which modulates the permeability of the intestinal epithelial barrier by disassembling intercellular tight junctions (TJ). Disruption of TJ is associated with neonatal necrotizing enterocolitis (NEC). It has been shown bifidobacterium could protect the intestinal barrier function and prophylactical administration of bifidobacterium has beneficial effects in NEC patients and animals. However, it is still unknown whether the zonulin is involved in the gut barrier dysfunction of NEC, and the protective mechanisms of bifidobacterium on intestinal barrier function are also not well understood. The present study aims to investigate the effects of bifidobacterium on intestinal barrier function, zonulin regulation, and TJ integrity both in LPS-induced enterocyte barrier injury of Caco-2 monolayers and in a rat NEC model. Our results showed bifidobacterium markedly attenuated the decrease in transepithelial electrical resistance and the increase in paracellular permeability in the Caco-2 monolayers treated with LPS (*P* < 0.01). Compared with the LPS group, bifidobacterium significantly decreased the production of IL-6 and TNF-α (*P* < 0.01) and suppressed zonulin release (*P* < 0.05). In addition, bifidobacterium pretreatment up-regulated occludin, claudin-3 and ZO-1 expression (*P* < 0.01) and also preserved these proteins localization at TJ compared with the LPS group. In the in vivo study, bifidobacterium decreased the incidence of NEC from 88 to 47% (*P* < 0.05) and reduced the severity in the NEC model. Increased levels of IL-6 and TNF-α in the ileum of NEC rats were normalized in bifidobacterium treated rats (*P* < 0.05). Moreover, administration of bifidobacterium attenuated the increase in intestinal permeability (*P* < 0.01), decreased the levels of serum zonulin (*P* < 0.05), normalized the expression and localization of TJ proteins in the ileum compared with animals with NEC. We concluded that bifidobacterium may protect against intestinal barrier dysfunction both in vitro and in NEC. This protective effect is associated with inhibition of proinflammatory cytokine secretion, suppression of zonulin protein release and improvement of intestinal TJ integrity.

## Introduction

Intestinal barrier dysfunction mainly refers to the abnormal increased intestinal permeability, which can make pathogens and foreign antigens cross the epithelial barrier[1]. Intestinal permeability is regulated by tight junctions(TJ) formed between intestinal epithelial cells (IEC) at the most apical areas of the epithelium. TJ form a selectively permeable intercellular barrier at the apical aspect of the enterocyte lateral membranes and regulate the paracellular movement of molecules between the intestinal lumen and subepithelial tissues[2]. Formation of functional TJ is critical for the maintenance of gut permeability and intestinal barrier function[3,4]. Several TJ proteins have been identified; among them, the transmembrane proteins occludin, claudins and cytoplasmic proteins zonula occludens 1(ZO-1) are considered crucial for creating the seal and regulating intestinal permeability[5]. Disruption of TJ is associated with a number of gastrointestinal diseases, including neonatal necrotizing enterocolitis (NEC). NEC is the most common and devastating gastrointestinal diseases of premature infants and is characterized by loss of intestinal barrier function[6–8].

Lipopolysaccharide(LPS), a component of the outer wall of Gram-negative bacteria, induces the barrier dysfunction by activating proinflammatory mediators (e.g., interleukin-6 (IL-6), tumor necrosis factor-α (TNF-α)), injuring epithelial cells and the TJ between epithelial cells[9]. It has also been reported that LPS-induced systemic inflammation leads to functionally significant alterations in the expression of key TJ proteins in the ileal epithelium[10]. Moreover, circulating LPS and proinflammatory cytokines were frequently found elevated in NEC patients, and its role in the alteration of the intestinal TJ barrier has been well established[11,12]. This suggests that both LPS and NEC-induced inflammation may impair TJ barrier, resulting in gut barrier dysfunction and abnormal increased intestinal permeability. However, the regulating mechanism of intestinal permeability is still incompletely understood. The newly discovery of zonulin protein, as the only physiological mediator known to regulate intestinal permeability by modulating intercellular TJ[13], led us to determine whether zonulin is involved in the regulation of intestinal barrier function in relation to NEC.

Zonulin is a eukaryotic analogue of the *Vibrio cholerae*–derived zonula occludens toxin (Zot), which regulates the intestinal epithelial paracellular permeability and modulates TJ protein expression and distribution through a protein kinase Cα (PKCα)-mediated actin polymerization[14]. Previous studies have shown that zonulin is over expressed in TJ dysfunction disorders in which TJ dysfunction is central, including celiac disease (CD) and type 1 diabetes (T1D)[15,16]. In the BioBreeding diabetic prone rat model of T1D, zonulin-dependent increases in intestinal permeability precede the onset of T1D by 2–3 weeks[15]. Moreover, the expression of zonulin in intestinal tissue and zonulin concentration in sera were increased in CD patients during the acute phase of CD[16]. These findings suggest that zonulin may play a key role in TJ dysfunction. Besides that pathogenic bacterial colonization has been identified as a powerful trigger to cause zonulin secretion in the intestine[17]. Thus, considering the central role of TJ dysfunction and bacterial colonization in the patheogenesis of NEC[18], the severe inflammatory disease such as NEC which colonized a large number inappropriate bacteria may has potential ability to induce zonulin release and it strongly revealed that zonulin protein might involved the pathological process of NEC by regulating intestinal permeability and modulating intercellular TJ.

Probiotics are living, nonpathogenic microorganisms that colonize the intestine and provide benefit to the host[19]. Bifidobacterium is predominating probiotic organisms in the intestinal flora of breast-fed infants while other obligate anaerobes are rare[20]. Studies both in human trails and animal studies has shown that bifidobacterium protects intestinal barrier function and prophylactic administration of bifidobacterium has beneficial effects in NEC[21–24]. However, the protective mechanisms of bifidobacterium on intestinal barrier function in NEC are not well understood yet. The aims of this study were to investigate the protective effects and underlying mechanisms of bifidobacterium on intestinal barrier function in LPS-induced enterocyte barrier injury of Caco-2 monolayers and in a rat NEC model. We evaluated the effect of bifidobacterium on intestinal barrier function, the underlying mechanisms about inflammatory cytokine production, zonulin regulation, and the expression and localization of TJ proteins were also determined.

## Materials and Methods

### Cell culture and treatment

Human Caco-2 cells (a generous gift from Dr. Gao Min, The Third Military Medical University, Chongqing, China) were obtained from the American Type Culture Collection (Manassas, VA, USA). Caco-2 cells were cultured in Dulbecco's Modified Eagle's Medium(DMEM) supplemented with 10% fetal bovine serum, 50 U/ml penicillin and 50 U/ml streptomycin (all from Invitrogen, USA). The cells were incubated in a humidified incubator with 5% CO_2_ at 37°C and passaged by routine trypinization. Cell viability was measured by the MTT method as previously described. The cells were plated at a density of 5x10^4^/cm^2^ on collagen-coated permeable polycarbonate membrane Transwell supports with 0.4 um pores(Corning, USA) and were grown as monolayers prior to the experiments.

In the LPS experiments (LPS group), the cell monolayers were treated with LPS (Sigma, USA) from *Escherichia coli* 055: B5diluted in ddH_2_O, used at 1.0ug/ml for 24h, and LPS was added to the basolateral side of Transwells. In the vehicle experiments, the ddH_2_O was injected into the untreated cells as vehicle group. In the bifidobacterium experiments(LPS+BIF group), The Caco-2 cells were treated with LPS (treated the same way as before) and bifidobacterium for 24h, and approximately 10^8^CFU of bifidobacterium were added to apical side of Transwells. In here, the bifidobacterium(Microbial Library, Guangzhou, China) was inoculated in Luria broth and cultured in anaerobic jar containing 90% N_2_ and 10% CO_2_ at mosphere at 37°C for 48-72h. Then, bifidobacterium was centrifuged to pellet down the bacteria, washed with saline, and adjusted to 10^8^CFU/ml in saline by measuring the optical density at 600nm using spectrophotometer (Thermo, USA).

### In vitro TEER Assay

TEER values, an indicator of TJ permeability to ionic solutes, were measured at 1h, 3h, 6h, 12h after establishing Caco-2 cell monolayers using a Millicell-ERS voltohmmeter (Millipore,USA) according to the manufacturer’s instructions. The resistance value of the filters and fluids was be subtracted in each measurement when calculated.

### In vitro intestinal paracellular permeability assay

Barrier function was determined at 1h, 3h, 6h, 12h following treatments by apical to basolateral flux of 10-kDa fluorescein isothiocyanate-labeled dextran(FITC-dextran). The apical chamber was filled with FITC-dextran (0.5ml, 100ug/ml), and 2h later, the concentrations of FITC-dextran in the basolateral chamber were determined using a fluorometer (Thermo, USA) (excitation, 427 nm; emission, 536 nm). A fluorescein permeability(*P*) was calculated according to the following equation: *P*(%) = *BP*/*AP*, where *BP* is the fluorescein concentrations of the basolateralchamber, *AP* is the apical chamber concentration.

### Electron microscope of cells

The cells samples were fixed in 3% glutaraldehyde and postfixed in 1% osmium tetroxide. Cells were scraped with a rubber scraper and spun at 5,000rpm for 4 min. Samples were dehydrated through Ethanol and then into propylene oxide and a 1:1 propylene oxide-Eponate mixture and left overnight, capped, at room temperature. The samples were polymerized at 70°C for 48 h. Thin sections were cut using a diamond knife and stained with 5% uranyl acetate for 15 min. The sections were viewed and photographed in an electron microscope (Hitachi, Japan).

### Enzyme-linked immunosorbent assays (ELISA)

The release of the zonulin proteins in the Caco-2 cells and in serum of animals were determined with ELISA (HuShang Biological Technology Co, China). Briefly, the Caco-2 cells were treated with LPS and LPS+BIF for 24 h, cellfree supernatants were collected and centrifuged at 4°C, 1000g for 15min, then the supernatants were obtained for preparation. The rat blood was centrifuged at 4°C, 3000g for 10min, and the seurm was collected. The absorbance of the samples (450 nm) was measured using a microplate (BioTek, USA) according to the manufacturer’s instructions. All samples were tested in duplicate.

The production of IL-6 and TNF-α in Caco-2 cells and in the intestinal tissue of rat pups were determined with ELISA kits purchased from Wuhan USCN Life Science Inc. The rat intestinal tissues were homogenized on ice in NP40 lysis buffer(Beyotime Biotechnology, China), and the homogenates were quantified using the BCA assay(Beyotime Biotechnology, China). Cell supernatants and tissue homogenates were collected respectively for the determination of IL-6 and TNF-α concentrations according to the manufacturers’ instructions.

### Western blot analysis

For tissue lysate preparation, 2-cm distal intestine(ileum) was homogenized with a hand-held homogenizer in lysis buffer. For cell culture lysate preparation, cells on plates were rinsed with cold PBS and lysed in the same lysis buffer used for tissue lysate preparation. Total protein were extracted from the ileum tissue and Caco-2 cells respectively (keyGEN BioTECH, China). Protein concentrations were determined by Pierce BCA assay. Proteins were loaded on polyacrylamide gels and the proteins in the gels were transferred to PVDF membrane (Bio-Rad, USA). For immunodetection the following antibodies were used: anti-claudin-3, anti-occludin and anti -ZO-1(1:400, Invitrogen, USA), and then incubated with an HRP-conjugated anti-rabbit secondary antibody(1:2000, Cell Signaling Technology, USA) (room temperature, 1h). The protein bands were visualized with a G-BOX imaging system(Syngene, UK) using an ECL assay kit (Pierce, USA).

### Immunofluorescence staining of cells

Cells were grown in chamber slides. After washing with PBS (3×5min), the cells were fixed with 3.7% paraformaldehyde/PBS for 10min at room temperature. Cells were permeabilized with 0.1% TritonX-100 in 1% BSA/PBS (room temperature, 30 min) and then were blocked with 10% BSA/PBS (room temperature, 1 h). After washing with PBS (3×10min), the cells were incubated with claudin-3, occludin and ZO-1 antibodies (Invitrogen, USA) at 1:50 dilution overnight at 4°C. After several washes, the slides were incubated again for 45 min with appropriate secondary FITC-conjugated antibodies (Sigma, USA). Nuclei were counter stained with diamidino-2- phenylindole(DAPI) (Sigma, USA). The slides were imaged using a fluorescent microscope (Nikon, Japan).

### Immunohistology of tissues

Briefly, after deparaffinization and rehydration, antigen unmasking was achieved using 10mmol/L citric acid buffer for 20 min, followed by blocking of endogenous peroxidase using 0.1% hydrogen peroxide for an additional 10min. And then the specimens were incubated with rat polyclonal anti-rat Claudin-3, Occludin, ZO-1(Invitrogen, USA), overnight at 4°C in a moist incubation chamber. After washing three times in PBS, slides were incubated with the biotinylated secondary antibody against the rabbit polyclonal antibody produced in goat(Zhongshan goldenbridge, China). The Vectastain Elite ABC reagent(Zhongshan goldenbridge, China) was applied for 30 min, slides were washed three times with PBS. Sections were then counter stained with hematoxylin for 20 seconds, dehydrated, and cover slipped. Sections from all three experimental groups were stained for a specific primary antibody at the same time, so that comparisons between groups could be assessed. The immunostained sections were analyzed under optical microscope(Olympic, Japan).

### Ethics statement

The animal studies were conducted in accordance with the Guide for the Care and Use of Laboratory Animals of the National Institutes of Health. All animal experimental procedures in this study were approved by the Ethics Committee of Chongqing Medical University(Permit Number: SYXK2007-0016). According to Institutional Animal Care and Use (IACUC)-approved procedures, the euthanasia is necessary when animal meet one of the criterion, including (1) rapid weight loss of 20 percent within three days, (2) inappetence, (3) moribund state, and (4) signs of severe organ system dysfunction(such as inactivity, unaware of stimuli, skin pallor and cyanosis) and non-responsive to treatment, or with a poor prognosis as determined by a veterinarian. Health checks were done four times a day at a minimum to assess the healthy state of animals. The rat was euthanized by decapition at the end of experiment or one of the criterion that described above-mentioned. Efforts were made to minimize animal suffering and to reduce the number of animals used. The rat NEC model was established in neonatal Sprague-Dawley (SD) rats by hypoxia-cold stress, formula feeding and LPS treatment. In the entire modeling process, there is no surgical procedures that cause pain or discomfort in animals. Considering the breath problem caused by hypoxia, we wound timely administer oxygenation treatment to reduce animal suffering. All rats were housed on a 12/12 hour light/dark cycle with a moderate temperature (23±2°C). The investigators were as gentle as possible to the rat pups. After getting stressed, the rat pups were put into the incubator to keep warm rapidly.

### NEC model establishment and treatment

All experimental Sprague-Dawley(SD) rats (SPF grade) were obtained from the Animal Experiment Center of Chongqing Medical University. The rat NEC model was established as previously described[25]. Briefly, neonatal SD rats were collected by caesarian section 24 h before the expected date birth. At postnatal day 1, the SD rat pups(6–8 g) were hand-fed every 4h using a silicone rubber tube (0.2 mm) with 0.1mL of cow’s milk-based rat milk substitute formula. Later, the feeding amount was increased 0.1mL every 24 hours until 0.3mL. The rat-milk substitute was based on the Auestad[25] reported formula. Meanwhile rat pups got stressed twice every day with hypoxic-reoxygenation treatment (breathing 100% nitrogen gas for 3min then 100% oxygen gas for 3min immediately) followed by a cold stress (4°C for 10min) for 3 days. All pups were fed with LPS at 5mg/kg dissolved in 0.1mL sterile water via intragastric tube once a day for 3 days to induce NEC.

The rat pups were divided into three groups (n = at least 20 in each group): healthy control group, NEC group and BIF group. In the BIF experiments, pups were treated with formula containing 1×10^8^CFU per day of bifidobacterium when being submitted to the NEC protocol. Naturally born and neonatal rats fed from dams were included in experiments as healthy controls.

### Histology and NEC evaluation

The intestines were collected and fixed in 10% paraformaldehyde and embedded in paraffin. Hematoxylin-Eosin (H&E) staining were performed and used to assessed histologically for ileal damage according to the NEC scoring system as previously published. Histological changes in the ileum were evaluated and graded by a blinded evaluator as follows [25]: 0 (normal) no damage; 1 (mild), slight submucosal and/or lamina propria separation; 2 (moderate), moderate separation of the submucosa and/or lamina propria and/or edemain the submucosa and muscular layers; 3 (severe), severe separation of the submucosa and/or lamina propria and/or severe edema in the submucosa and muscular layers with regional villous sloughing; and 4 (necrosis), that is loss of villi and necrosis. To determine the incidence of NEC, animals with histological scores of less than 2 were considered to not have developed NEC, whereas animals with histological scores of 2 or greater were considered to have developed NEC.

### Barrier function permeability assay in vivo

This measure is based on the intestinal permeability towards 4000Da fluorescent dextran–FITC (DX-4000–FITC) as described[26]. Briefly, rat that had fasted for 8h were given DX-4000–FITC (Sigam, USA) by gavage (40 mg/100g body weight, 20 mg/mL). After 4h, the blood was collected from the heart and centrifuged at 4°C, 3000g for 10min. Plasma was diluted in an equal volume of PBS (pH 7.4) and analyzed for DX-4000–FITC concentration with a fluorescence spectrophotometer (Thermo, USA) at an excitation wavelength of 480nm and emission wavelength of 520nm. Standard curves were obtained by diluting FITC–dextran in non-treated plasma diluted with PBS (1:3 v/v).

### Statistical analysis

The statistical analyses were performed with SPSS software version 19.0. The data are presented as the means ± standard deviation. A normality test and a homogeneity test for variance were performed first. If the data were in compliance with a normal distribution and homogeneity of variance, an ANOVA with Bonferroni’s post-test or a Student’s t-test was performed; otherwise, a rank sum test was used.The categorical data were analyzed using the Chi-squared test. Correlation analyses were performed using the Pearson’s test. *P*<0.05 was considered to be statistically significant.

## Results

### 1. Bifidobacterium reduced the severity and incidence of NEC in a rat NEC model

#### 1.1 Survival rates of NEC

The survival rates for experiment animals were as follows: control, 20/20; NEC, 25/36; and BIF, 32/36. Animals with NEC had a significantly lower survival rate compared with control animals (P<0.05).

#### 1.2 Body weights

**Fig 1:**
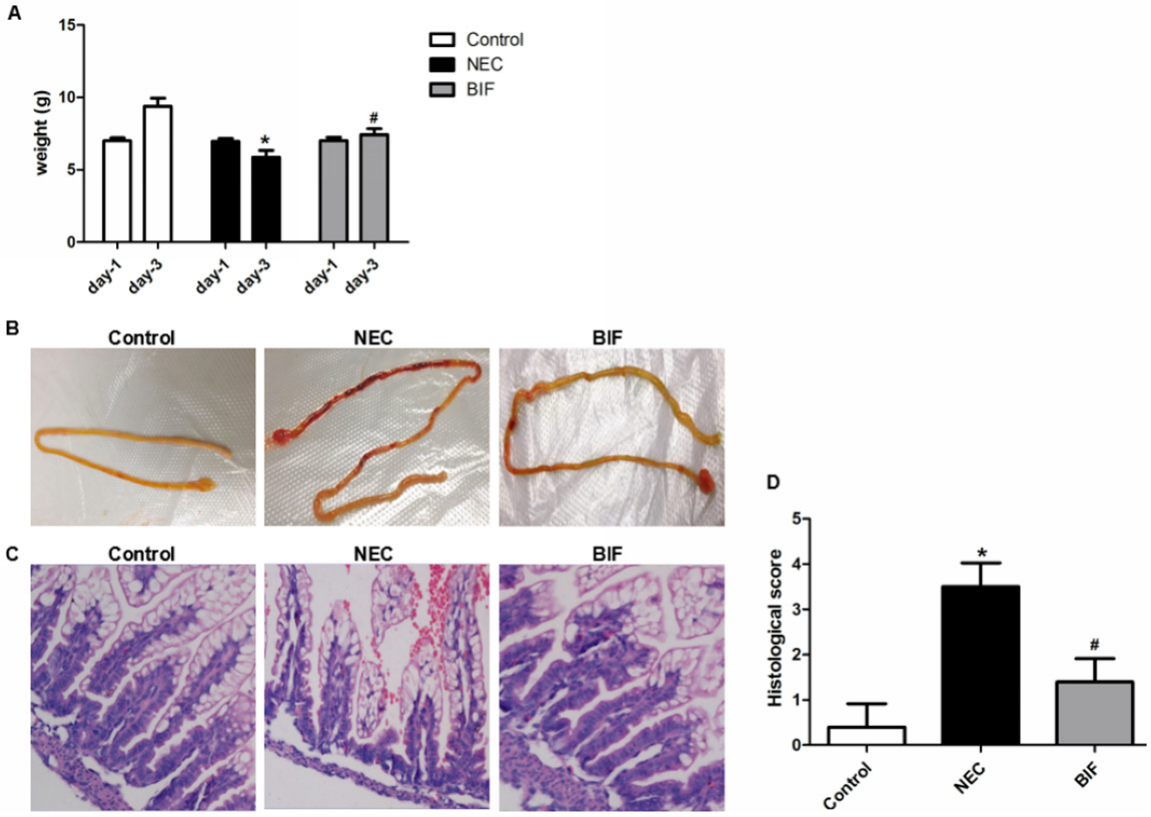
Bifidobacterium reduced the severity and incidence of NEC in a rat NEC model. **(A)** Body weight changes. *P < 0.01 vs the control group, ^#^*P* < 0.01 vs the NEC group, n = 9–11 animals per group. Three independent experiments were performed in duplicate. **(B)** Macroscopic appearance of the gastrointestinal tract. In rat pups with NEC, dilation, significant hemorrhage, and discoloration were seen in the terminal ileum. **(C)** Images of H&E staining using light microscopy. The histological changes in the terminal ilea (representative images) in the control, NEC and BIF groups. Magnification: ×20. **(D)** Intestinal histological score. *P < 0.01 vs the control group, ^#^*P* < 0.01 vs the NEC group, n = 6 animals per group.

The basal body weight of rat pups was comparable before the start of the study. Control group rat pups showed obviously increased body weights from day-1 to day-3. However, the body weights in the NEC group were dramatically lower (day-3: P<0.01) than those of the control group. Although BIF group rat pups showed increased body weights on day-1 to day-3, the body weights were still significantly lower (day-3: P<0.01) than those of the control group (Fig 1A).

#### 1.3 Macroscopic appearance of the small intestine

The neonatal rat model of NEC has similar clinical and pathological changes found in NEC patients. the abdomen is distended, blood could detected in stool and the most affected parts are ileum and proximal colon. Fig 1B shows the macroscopic appearances of the small intestine in control animals, NEC animals and BIF animals separately. Control pups did not exhibit macroscopic damages, whereas NEC animals showed obvious injury in the distal ileum, with dilation, severe hemorrhage and discoloration. Ileal damages in rat pups administered bifidobacterium were significantly reduced compared with NEC rat pups (Fig 1B).

#### 1.4 H&E staining, Intestinal histological score and Incidence of NEC in a rat NEC model

Intestinal sections were H&E-stained and scored as described under “Materials and Methods”. HE staining of the small intestine showed the short and ruined mucosa compared with that of control group rat pups (Fig 1C). However, BIF moderated the ileal damage of rats intestinal morphology which was examined by light microscopy using a scoring system from 0 to 4 to estimate the severity of NEC. Results from these measurements are shown in Fig 1C. Median intestinal histological score in animals receiving BIF was statistically markedly decreased from 3.64±0.49 in the NEC group to 1.82±0.61 (P<0.01)(Fig 1D). The rats of control group showed no or mild abnormalities in ileal structure. In addition, the incidence of NEC was significantly reduced from 88% (22/25) in NEC group to 47% (15/32) in the BIF group.

### 2. Bifidobacterium protected intestinal barrier function in vitro and in a rat NEC model

#### 2.1 Evaluation of intestinal barrier function in vitro

**Fig 2:**
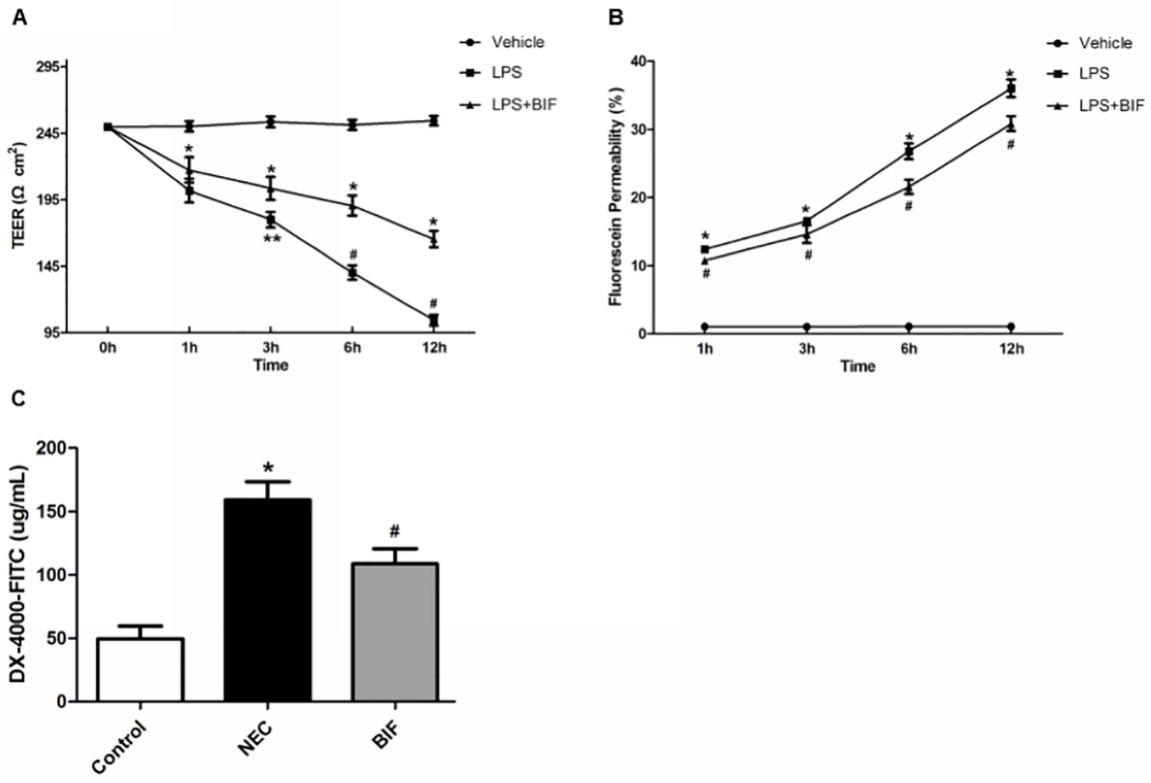
Bifidobacterium protected intestinal barrier function in vitro and in a rat NEC model. **(A)** The transepithelial electrical resistance (TEER) was tested at different time points. *P < 0.01 vs the vehicle group, ^#^*P* < 0.01, **P < 0.05 vs the LPS group. Values are mean ± SD from four independent experiments performedin duplicate. **(B)** Intestinal permeability was assessed in vitro using the fluorescein permeability (P) test at different time points, *P < 0.01 vs the vehicle group, ^#^*P* < 0.01 vs the LPS group. **(C)** The intestinal barrier function in vivo was evaluated by fluorescent dextran–FITC. *P < 0.01 vs the control group, ^#^*P* < 0.01 vs the NEC group, n = 6 animals per group.

It has been demonstrated that LPS caused intestinal barrier dysfunction. To investigate the effects of bifidobacterium on intestinal barrier function, we used a vitro model in which Caco-2 epithelial cell monolayers were treated with LPS. TEER is an indicator of epithelial paracellular permeability to ionic solutes and was used to assess intestinal barrier function. Compared with the vehicle-treated cells (untreated cells), TEER was consistently decreased in the LPS-induced cells at 1h, 3h, 6h and 12h and reached a lower level at 12h of LPS induction, indicating the disruption of the barrier function in the monolayers (Fig 2A). However, bifidobacterium significantly inhibited the reduction in TEER induced by treatment with LPS at all the above time point (Fig 2A).

Consistent with the changes in TEER, permeability(P) for FITC-dextran flux, an indicator of epithelial paracellular permeability to uncharged macromolecules, markedly increased when the Caco-2 monolayers were treated with LPS at 1h, 3h, 6h, and 12h (Fig 2B), with a higher increase at 12h of LPS induction. This indicated that the paracellular permeability to non-ionic macromolecules was increased by treatment with LPS. As shown in Fig 2B, treatment with bifidobacterium significantly reduced the increase in the paracellular FITC-dextran flux induced by LPS from 1h to 12h. These data suggest that treatment with bifidobacterium attenuates intestinal epithelial barrier dysfunction induced by LPS in vitro.

#### 2.2 Evaluation of intestinal barrier function in a rat NEC model

To determine whether bifidobacterium protection against NEC was associated with preservation of intestinal barrier function in the face of NEC stress, paracellular intestinal permeability was assessed by oral administration of the fluorescent tracer FITC-dextran (4 kDa) to all surviving pups at the end of the NEC animal experiment. The concentrations of fluorescent FITC-dextran in the blood were then measured as a reflection of intestinal permeability in vivo. As shown in Fig 2C, the healthy control group had the lowest level of FITC-dextran in the blood (better barrier function), and the untreated NEC experimental group had the highest levels of FITC-dextran in the blood (poorer barrier function) (Fig 2C). Bifidobacterium treatment in NEC animals significantly decreased FITC-dextran flux to blood to near healthy control levels, indicating that the supplementation of bifidobacterium protected barrier function.

### 3. Bifidobacterium reduced inflammatory response in vitro and in a rat NEC model

#### 3.1 Evaluation of inflammatory response in vitro

**Fig 3:**
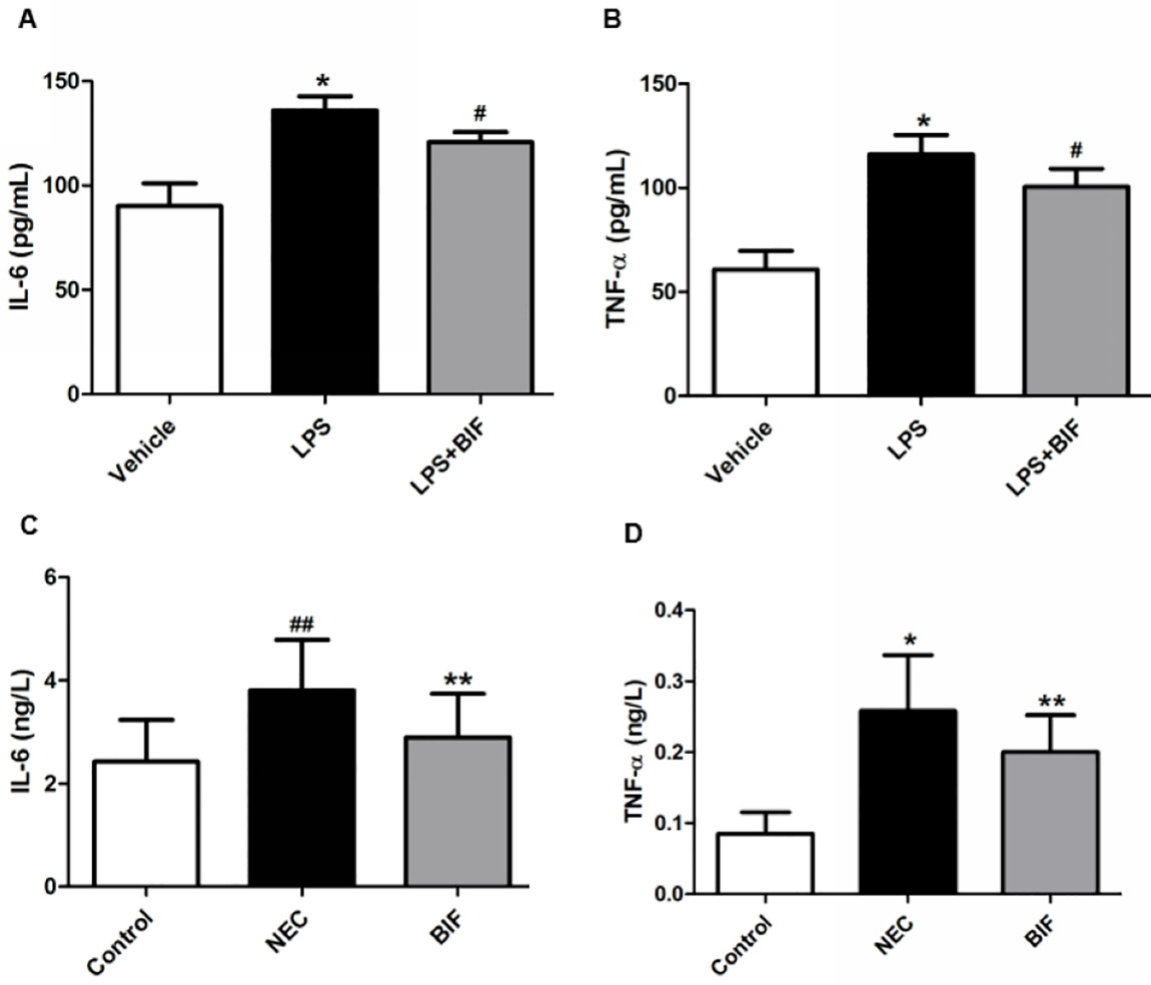
Bifidobacterium reduced inflammatory response in vitro and in a rat NEC model. **(A)** IL-6 and **(B)** TNF-α concentrations in the Caco-2 cells were determined by ELISA. three independent experiments were performed in duplicate. *P < 0.01 vs thevehicle group, ^#^*P* < 0.01 vs the LPS group. **(C)** IL-6 and **(D)** TNF-α concentrations in rat NEC model. *P < 0.01, ^##^*P* < 0.05 vs the control group, **P < 0.05 vs the NEC group, n = 5 animals per group. Three independent experiments were performed in duplicate.

We next assessed whether bifidobacterium protects cells by influencing the secretion of inflammatory cytokines in vitro. As shown in Fig 3A and 3B, stimulation with LPS resulted in significant increases in the production of IL-6 and TNF-α compared with vehicle-treated cells (p < 0.01, Fig 3A and 3B). However, the production of both inflammatory cytokines, IL-6 and TNF-α, which elevated with LPS, were significantly decreased following pretreatment with bifidobacterium (*P* = 0.005, *P* = 0.009, Fig 3A and 3B).

#### 3.2 Evaluation of inflammatory response in the ileum of rat NEC model

Cytokines are key regulators in inflammation and several cytokines are dysregulated in NEC(58). Proinflammatory IL-6 and TNF-α are the major cytokines associated with NEC pathogenesis and neonatal sepsis. To evaluate whether the protective effects of bifidobacterium were mediated via the inhibition of cytokine secretion, the temporal secretion profiles of IL-6 and TNF-α were investigated in this newborn rat NEC model. The ELISA results showed that the secretion of IL-6 and TNF-α was significantly decreased in the BIF-treated rats compared to the rats with NEC (*P* = 0.028, *P* = 0.032, Fig 3C and 3D).

### 4. Bifidobacterium decreased zonulin protein release in vitro and in a rat NEC model

#### 4.1 Evaluation of zonulin protein release in vitro

**Fig 4:**
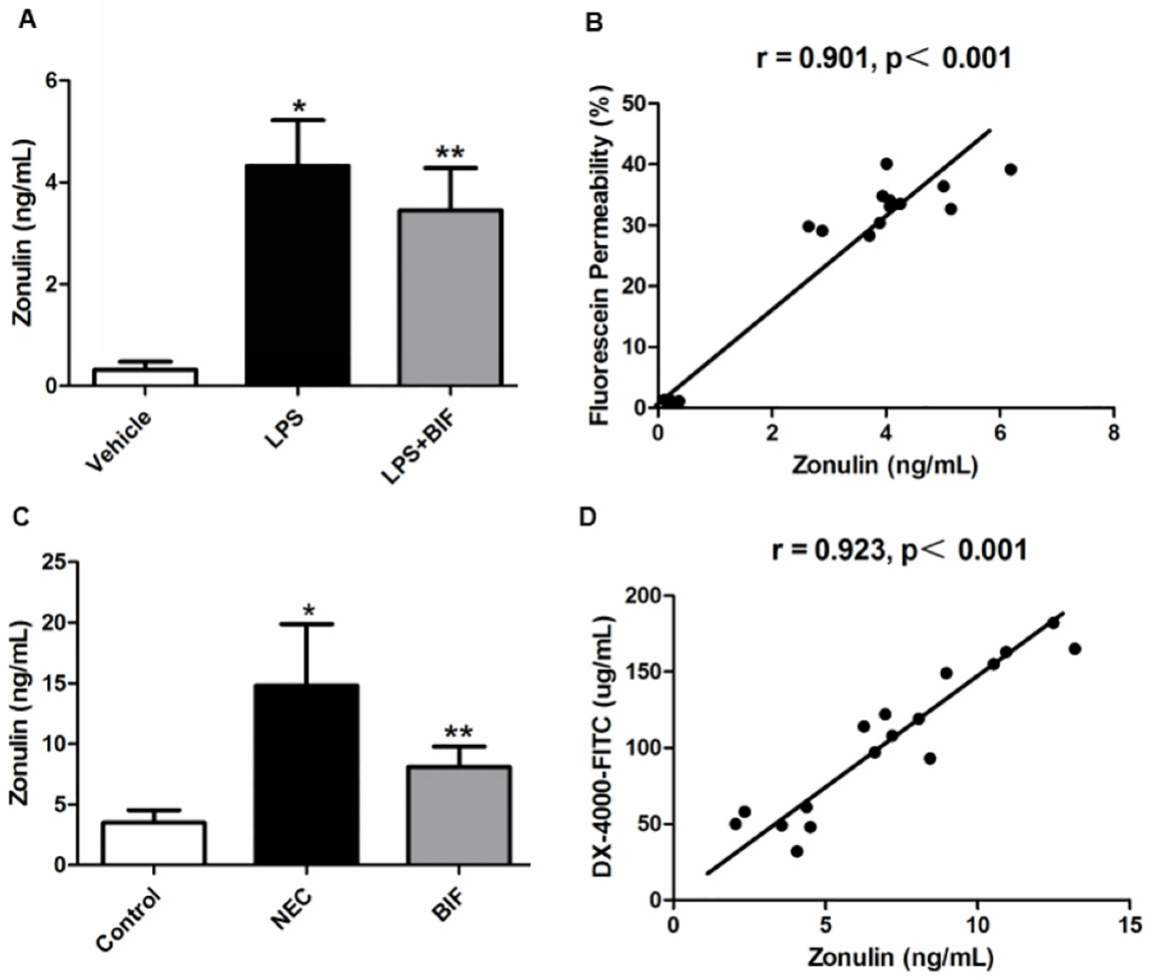
Bifidobacterium decreased zonulin protein release in vitro and in a rat NEC model. **(A)** The release of zonulin was determined by ELISA in the Caco-2 cells. Data are mean ± SD from six independent experiments. *P < 0.01 vs the vehicle group, **P < 0.05 vs the LPS group. **(B)** Correlation analysis between fluorescein permeability and the release of zonulin in the Caco-2 cells. **(C)** The level of serum zonulin of animals was determined by ELISA. *P < 0.01 vs the control group, **P < 0.05 vs the NEC group, n = 6 animals per group. **(D)** Correlations analysis between intestinal permeability markers (plasma DX-4000–FITC) and serum zonulin concentrations.

Zonulin protein is an important modulator of intestinal permeability and intercellular TJ. As the above results showed that intestinal permeability was reached a rather higher level at 12h in Caco-2 epithelial cell monolayers after treatment of LPS (Fig 2B). Therefore, to determine whether the protective role of bifidobacterium on intestinal barrier function is involved in zonulin protein release in vitro, we choose the time point of 12h for testing zonulin release, and the correlation analysis between intestinal permeability and zonulin was also be analyzed at 12h. Our results showed that the levels of zonulin in the LPS-treated cells was significantly greater than in the vehicle-treated cells(p < 0.01, Fig 4A); However treatment with bifidobacterium significantly reduced the increase in the zonulin release induced by LPS(p < 0.05, Fig 4A). Furthermore, There was a significant positive correlation between high zonulin levels and increased intestinal permeability(p < 0.001) (Fig 4B). These results suggest that bifidobacterium-mediated protection against intestinal barrier dysfunction in vitro may be regulated via zonulin.

#### 4.2 Evaluation of zonulin protein release in a rat NEC model

NEC is a disease involving the major risk factor of inappropriate intestinal bacterial colonization and characterized pathologically by increased intestinal permeability. To determine whether the protective effect of bifidobacterium on intestinal barrier function of NEC is mediated by zonulin. The blood concentration of zonulin was measured using ELASA assay. As shown in Fig 4C, the serum zonulin was significantly different between the control group and NEC group (3.48 ± 1.05 ng/mg protein vs 13.31 ±5.11 ng/mg protein, P<0.01). The serum zonulin concentration in the BIF group (8.40 ±2.27 ng/mg protein) was significantly lower than in the NEC group (P = 0.01, Fig 4C). These results indicated that the serum zonulin was increased in NEC, and BIF could lower the zonulin level efficiently. Moreover, serum zonulin levels and plasma DX-4000–FITC were positively and significantly correlated (r = 0.923, *P*＜0.001, Fig 4D).

### 5. Bifidobacterium prevents the disruption of TJ in vitro and in a rat NEC model

#### 5.1 Changes in expression and localization of TJ in vitro

**Fig 5:**
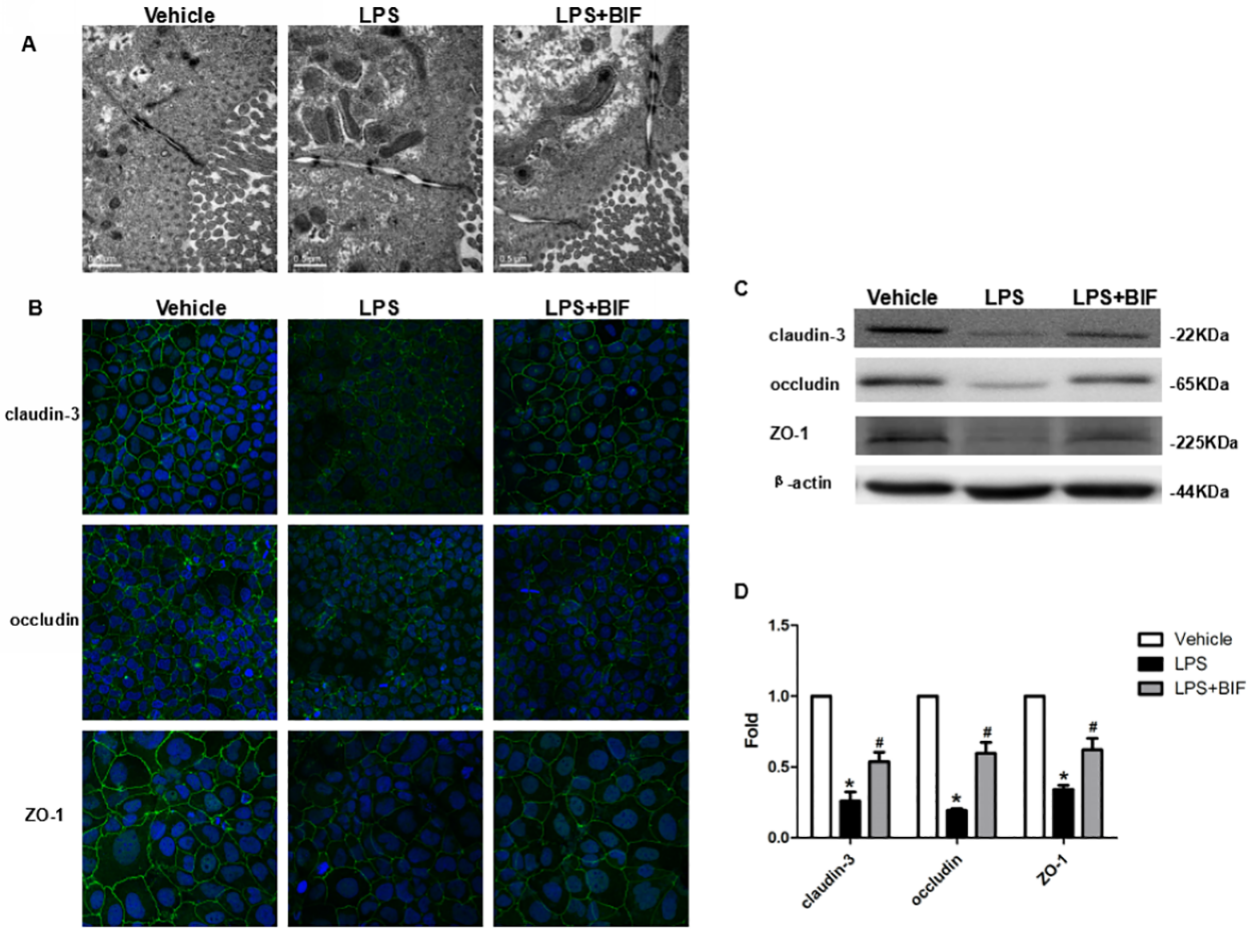
Bifidobacterium prevented the disruption of TJ in vitro. **(A)** Electron micrographs reveal the changes of intact TJ in vehicle group, LPS group and LPS+BIF group. **(B)** Immunofluorescence staining of TJ proteins localization in Caco-2 cells with or without LPS and BIF. Magnification: ×40. **(C)** Western blot for TJ proteins. Caco-2 cells were grown and treated with LPS and BIF and lysed. The lysates were used for immunoblotting for claudin-3, occludin, ZO-1 and β-actin. Representative results of one experiment are shown. Similar results were obtained in three independent experiments: vehicle group, LPS group, LPS+BIF group. **(D)** The intensity of the bands was quantified by scanning densitometry, standardized with respect to β-actin protein and expressed as mean ± SD fold change compared with vehicle cells. *P < 0.01 vs the vehicle group, ^#^*P* < 0.01 vs the LPS group.

Increased intestinal permeability in the absence of histological evidence of epithelial damage suggests a TJ defect. We therefore examined enterocyte TJ ultrastructure. At 24 hours, both vehicle treated cells, LPS treated cells and LPS+BIF treated cells exhibited adjacent membrane kisses, which are typical of intact TJ structures (Fig 5A). The apical-to-basolateral TJ length was greater in LPS treated cells at 24 hours compared with vehicle treated cells, and LPS+BIF treated cells consistent with TJ restructuring (Fig 5A).

The changes in the expression of TJ proteins, major component of the TJ, were examined to investigate LPS-induced barrier injury. In addition to barrier protection, BIF treatment prevented LPS-induced up-regulation of claudin-3, occludin, ZO-1 (Fig 5B). In vehicle-treated cells, Immunofluorescence microscopy exhibited a normal, organized structure and well characterized TJ proteins localization at the cell boundaries, while the LPS-treated cells exhibited a disordered structure and fainter and more diffuse staining at the places (Fig 5B). In BIF+LPS-treated cells, We observed stronger staining both on the membrane and in the cytoplasm, compared with cells treated with LPS(Fig 5B). These results show that BIF was able to protect TJ protein expression as well as subcellular distribution from LPS-induced damages in Caco-2 monolayers.

The Western blot results demonstrated that the expression of claudin-3, occludin, ZO-1 proteins were all dramatically decreased in the cells treated with LPS (P<0.01) compared to the cells treated with vehicle (Fig 5C and 5D); whereas, the expression of these TJ proteins were significantly increased in the cells treated with LPS+BIF(P<0.01) compared to the cells treated with LPS(Fig 5C and 5D).

#### 5.2 Changes in expression and localization of TJ in the ileum of rat NEC model

Immunohistochemical staining of TJ proteins revealed significant morphological changes and subcellular localization. As previously suggested, a representative immunohistochemical assay performed on intestinal sections of rats demonstrated an intact network of Claudin-3, Occludin and ZO-1 proteins which were predominantly localized along the apical cellular border. Immunohistochemical microscopy revealed that claudin-3 was localized predominantly in the crypts. In animals with NEC, there was increased staining in the crypts near the apical membrane compared with control animals, BIF treatment normalized claudin-3 expression similar to control animals and redistributed of claudin-3 to the apical and basolateral membranes along the crypt-villus axis; ZO-1was mainly localized in the cell boundaries and in the cytoplasm along the crypt-villus axis in all groups with the least signal detected in the NEC group, whereas treatment with BIF significantly increased ileal ZO-1 expression to similar levels seen in control animals. Occludin was localized near the apical membrane in the crypts and in the cytoplasm along the villi and the expression was significantly decreased in the animals with NEC compared with control group. After BIF treatment, the expression and distribution of occludin were similar in control tissues.

**Fig 6:**
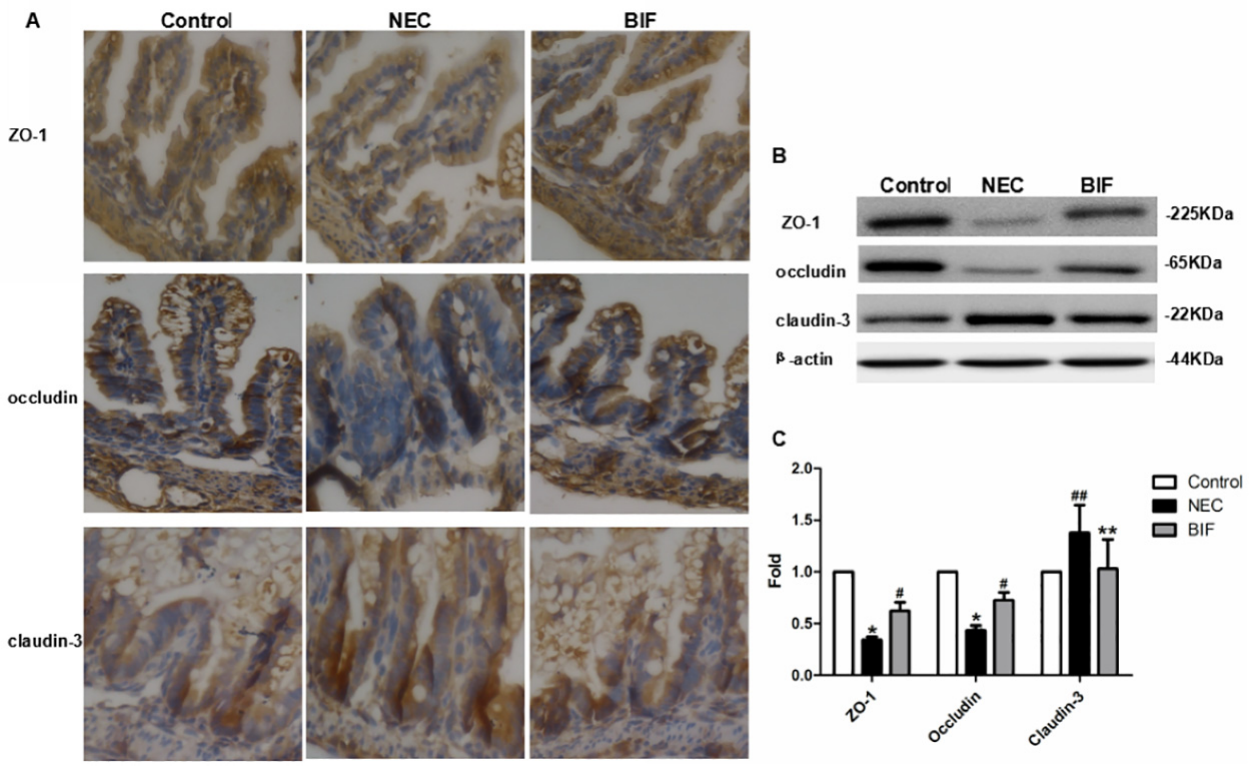
Bifidobacterium prevented the disruption of TJ in a rat NEC model. **(A)** TJ proteins localization was evaluated by Immunohistochemical staining in the terminal ileum of neonatal rats. Representative slides for control, NEC, and BIF were shown. Magnification: ×40, n = 3 to 6 per group. **(B)** Western blot for TJ proteins. Terminal ilea were subjected to immunoblotting for ZO-1, occludin claudin-3 and β-actin. Representative results of one experiment are shown. Similar results were obtained in three independent experiments: control group, NEC group, BIF group. **(C)** The intensity of the bands was quantified by scanning densitometry, standardized with respect to β-actin protein and expressed as mean ± SD fold change compared with control animals.*P < 0.01, ^##^*P* < 0.05 vs the control group, ^#^*P* < 0.01, **P < 0.05 vs the NEC group.

The expression of ZO-1, claudin-3 and occludin were evaluated by immunoblot of intestinal lysates from rat pups sacrificed at the end of the animal experiments. We observed that the expression of ZO-1 protein and Occludin protein were obviously decreased in the NEC intestines compared with healthy controls (P<0.01 Fig 6B and 6C); claudin-3 in the NEC group were significantly increased compared with controls (P<0.05 Fig 6B and 6C). Interestingly, BIF had a divergent effect on the expression of these three TJ proteins with claudin-3 decreasing, ZO-1 and occludin increasing with BIF administration. Supplementation of BIF into formula resulted in normalization of ileal claudin-3, ZO-1 and occludin proteins expression to control levels.

## Discussion

NEC is a devastating disease of neonates and is associated with high morbidity and mortality[27,28]. Previous studies have shown that bifidobacterium protects the intestinal barrier function and oral administration of bifidobacterium has beneficial effects of NEC[21–24]. However, the protective mechanisms of bifidobacterium on intestinal barrier function in NEC are not well understood. In the present study, we used a Caco-2 monolayers and a rat NEC model to investigate the protective effects of bifidobacterium on intestinal barrier dysfunction in vitro and in NEC. Our present study showed that bifidobacterium reduced the incidence and severity in rat NEC model. It also attenuated LPS-induced enterocyte barrier injury of Caco-2 monolayers as well as intestinal barrier dysfunction in NEC rats. Mechanisms of bifidobacterial-mediated protective effect on intestinal barrier function in vitro and in NEC include the inhibition of proinflammatory cytokine secretion, the suppression of zonulin protein release, and the maintenance of TJ integrity.

The intestinal epithelium provide a physical barrier that protects the host against the unwelcome intrusion of micro-organisms[29]. The failure of intestinal barrier function induced with LPS resulted in an increase in mucosal permeability, which was hypothesized to be a major promoter of bacterial translocation and inflammatory cytokines release[30,31]. IL-6 and TNF-α are the critical cytokines involved in inflammation, and bifidobacterium is regarded as a common treatment strategy for information-related diseases, including NEC [22,32,33]. In our LPS- induced monolayers inflammation model and NEC rats, an increase in the production of IL-6 and TNF-α was shown to precede the intestinal barrier dysfunction, while bifidobacterium protected intestinal epithelial barrier function and significantly decreased the production of IL-6 and TNF-α both in Caco-2 monolayers and in a rat NEC model. This findings suggest that NEC is associated with overexpression of proinflammatory cytokines which not only induce intestinal inflammatory response but also lead to barrier disruption, and bifidobacterium preserve intestinal barrier function by inhibiting inflammatory cytokines release.

Intestinal barrier function and intestinal permeability are primarily determined by epithelial TJ[34]. The TJ is localized on the apical membrane of polarized epithelial cells and functions to create a barrier to the paracellular movement of solutes[2]. The TJ is a dynamic structure and can be disassembled and reorganized in response to various intracellular and extracellular stimuli, such as pathogenic bacteria, LPS, inflammatory mediators[35]. It has been demonstrated that disruption of TJ and loss of barrier function are associated with several conditions and diseases[36–39]. In colonic biopsies from patients with ulcerative colitis, reduced or redistributed of claudin-3 and -4 association with increased permeability has been reported[36]. In the IFN-γ-induced damages of T84 enterocytes, IFN-γ disruption of barrier function was associated with down-regulation of the TJ protein ZO-1, and decreased ZO-1 expression alone was sufficient to cause barrier dysfunction[39]. In our vitro study, we demonstrated that stimulation of LPS resulted in a significant decrease in the expression of occludin, claudin-3 and ZO-1. Moreover, normal distribution of occludin, claudin-3 and ZO-1 proteins was also altered in LPS-treated Caco-2 monolayers, leading to increased permeability of the intestinal barrier. Based on these data, we detected the expression and distribution of TJ proteins in a rat NEC model to determine their role in the impairment of intestinal barrier function. As shown in our vivo study, similar expression and distribution of ZO-1 and occludin was observed in NEC group, and claudin-3 distribution was less organized at the plasma membrane and localized in the cytoplasm throughout the villi in animals with NEC. Interestingly, claudin-3 expression was significantly increased in NEC group compared with the control group. Our findings are consistent with the study by Clark *et al*[5] in which increased claudin-3 expression was also observed in NEC intestines and correlated with the degree of NEC injury. As the claudins are thought to be the pore-forming proteins that regulate the size selectivity of the TJ barrier and contribute to increased paracellular permeability[40]. we speculate that the presence of claudin-3 in the crypts may promote a “leakier” epithelium, allowing for overall increased intestinal permeability.

Due to the similarity between Caco-2 monolayers and human intestinal epithelial cell barrier in morphology with the same cell polarity and TJ structure[41], we treated Caco-2 monolayers with bifidobacterium to determine whether the protective role in intestinal barrier is associated with the expression and distribution of TJ proteins. Our results showed that bifidobacterium could protect enterocyte barrier function via maintenance of occludin, claudin-3 and ZO-1 proteins levels and cellular localization in vitro in Caco-2 monolayers. Considering the immature epithelial cells do not polarize or form sufficient TJ to measure barrier function[40], we also measured the effect of bifidobacterium on TJ proteins and barrier function on immature intestinal epithelia under conditions of inflammatory stress in vivo in a rat NEC model. Our data in newborn rat pups demonstrated that bifidobacterium significantly attenuated intestinal barrier disruption, maintained TJ structure, preserved ZO-1, occludin protein and redistributed claudin-3 to the apical and basolateral membranes along the crypt-villus axis in vivo in an experimental NEC animal model. These findings imply that bifidobacterium could promote the recovery of NEC damaged TJ.

Recent studies have suggested that a newly discovery of the physiological protein zonulin which modulates the intestinal permeability by disassembling the intercellular TJ[13]. Although first reported zonulin, as a important modulator of intercellular TJ, involved in the pathogenesis of autoimmune disorders such as T1D and CD[15,16]. Several studies have indicated that mammalian small intestines react to the exposure to pathogenic enteric bacteria by activating the zonulin pathway, a system involved in the regulation of the small-intestinal TJ permeability[17,42]. Our in vitro data showed that the level of zonulin in LPS-treated Caco-2 monolayers was greater than that in vehicle-treated Caco-2 monolayers. These data are consistent with the clinic findings of Daniel *et al*[42] concerning sepsis patients, and this positive correlation between high zonulin level and increased intestinal permeability in Caco-2 monolayers further emphasizes the potential for zonulin to serve as a crucial modulator for intestinal permeability in NEC, a disease also involving the major risk factor of inappropriate intestinal bacterial colonization and characterized pathologically by increased intestinal permeability[43]. Indeed, in the present study, an elevated zonulin level, altered TJ proteins and increased intestinal permeability were observed in NEC rats compared with healthy controls. This first found data suggests that zonulin may play an important role in NEC via modulating intercellular TJ and consequently leading to increased intestinal permeability.

Probiotics are living, nonpathogenic microorganisms that colonize the intestine and provide benefit to the host[19]. There is strong evidence from both human trials and animal studies that the administration of probiotics protects against NEC [22–24]. However, the mechanisms remain poorly understood. In our vitro study, we found that bifidobacterium protects enterocyte barrier function and maintains TJ integrity in LPS-induced enterocyte barrier damage of Caco-2 monolayers. Unexpectedly, the release of zonulin was significantly decreased in Caco-2 monolayers after bifidobacterium treatment compared with LPS group. There is also accumulating evidence that bifidobacterium were shown to maintain integrity of intestinal mucosal barrier, reducing its permeability, and strengthening intestinal TJ. Based on the above evidence, we focused the bifidobacterium-mediated protection effect against intestinal barrier dysfunction of NEC on zonulin protein. Our results showed that zonulin release was markedly reduced in NEC rat pups after pretreatment with bifidobacterium and it was positively related to intestinal permeability, which revealed that zonulin protein might involve in the protective mechanisms of bifidobacterium on intestinal barrier function of NEC by regulating intestinal permeability and modulating intercellular TJ.

In conclusion, this study shows the protective effect of bifidobacterium on intestinal barrier function both in LPS-induced enterocyte barrier injury of Caco-2 monolayers and in a NEC rat model. The molecular mechanisms associated with these protective effects include inhibition of proinflammatory cytokine secretion, suppression of zonulin protein release and improvement of intestinal TJ integrity. Importantly, zonulin may be a crucial factor by which bifidobacterium protects intestinal barrier function.

## Supporting Information

S1 File The original data of this study. (XLS)

## Abbreviations

NEC: neonatal necrotizing enterocolitis
LPS: lipopolysaccharide
BIF: bifidobacterium
TJ: tight junctions
IEC: intestinal epithelial cells
ZO-1: zonula occludens 1
TLR4: toll like receptor 4
Zot: zonula occludenstoxin
PKCα: protein kinase Cα
CD: celiac disease
T1D: type 1 diabetes
DMEM: Dulbecco's Modified Eagle's Medium
TEER: transepithelial electrical resistance
FITC-dextran: fluoresce inisothiocyanate-labeled dextran
SD: Sprague-Dawley
H&E: Hematoxylin and Eosin
ELISA: enzyme-linked immunosorbent assay
PBS: phosphate-buffered saline
